# Contribution of IL-12/IL-35 Common Subunit p35 to Maintaining the Testicular Immune Privilege

**DOI:** 10.1371/journal.pone.0096120

**Published:** 2014-04-23

**Authors:** Hayato Terayama, Takayuki Yoshimoto, Shuichi Hirai, Munekazu Naito, Ning Qu, Naoyuki Hatayama, Shogo Hayashi, Kana Mitobe, Jun-ichi Furusawa, Izuru Mizoguchi, Takeshi Kezuka, Hiroshi Goto, Kaori Suyama, Hiroshi Moriyama, Kou Sakabe, Masahiro Itoh

**Affiliations:** 1 Department of Anatomy, Tokyo Medical University, Tokyo, Japan; 2 Department of Anatomy, Tokai University School of Medicine, Kanagawa, Japan; 3 Department of Immunoregulation, Institute of Medical Science, Tokyo Medical University, Tokyo, Japan; 4 Department of Ophthalmology, Tokyo Medical University, Tokyo, Japan; 5 Department of Anatomy, Showa University School of Medicine, Tokyo, Japan; Keio University School of Medicine, Japan

## Abstract

The testis is an organ with immune privilege. The comprehensive blood–testis barrier formed by Sertoli cells protects autoimmunogenic spermatozoa and spermatids from attack by the body’s immune system. The interleukin (IL)-6/IL-12 family cytokines IL-12 (p35/p40), IL-23 (p19/p40), IL-27 (p28/Epstein-Barr virus−induced gene 3 [EBI3]), and IL-35 (p35/EBI3) play critical roles in the regulation of various immune responses, but their roles in testicular immune privilege are not well understood. In the present study, we investigated whether these cytokines are expressed in the testes and whether they function in the testicular immune privilege by using mice deficient in their subunits. Expression of EBI3 was markedly increased at both mRNA and protein levels in the testes of 10- or 12-week-old wild-type mice as compared with levels in 2-week-old mice, whereas the mRNA expression of p40 was markedly decreased and that of p35 was conserved between these two groups. Lack of EBI3, p35, and IL-12 receptor β2 caused enhanced infiltration of lymphocytes into the testicular interstitium, with increased interferon-γ expression in the testes and autoantibody production against mainly acrosomal regions of spermatids. Spermatogenic disturbance was more frequently observed in the seminiferous tubules, especially when surrounded by infiltrating lymphocytes, of these deficient mice than in those of wild-type mice. In particular, p35-deficient mice showed the most severe spermatogenic disturbance. Immunohistochemical analyses revealed that endothelial cells and peritubular cells in the interstitium were highly positive for p35 at both ages, and CD163^+^ resident macrophages positive for p35 and EBI3, possibly producing IL-35, were also detected in the interstitium of 12-week-old mice but not those of 2-week-old mice. These results suggest that p35 helps in maintaining the testicular immune privilege, in part in an IL-35-dependent manner.

## Introduction

Because spermatogenesis begins during puberty, when immune tolerance already has been established, there are various autoimmunogenic materials in the testes that the body’s immune system recognizes as foreign [1]. To protect autoimmunogenic spermatozoa from attack by the immune system, the testes exhibit a distinctive form of immune privilege [1]–[3]. The blood–testis barrier (BTB), formed by tight junctions of Sertoli cells, partitions the interstitial blood compartment of the testis from the adluminal compartment of seminiferous tubules [4]. Previous studies demonstrated aspects of the immune privilege in the testes by using a local transplantation system [3], [5]. The testicular interstitium is resistant to polymorphonuclear cell infiltration, spermatic granuloma, vasculitis, and lymphangitis [3], [5]. Therefore, the testicular tissue outside the BTB, where many resident macrophages are normally present, is also protected from attack by the body’s immune system [3], [5]. To maintain the testicular immune privilege, the testicular cells express and secrete numerous immunoregulatory molecules, including androgens, macrophage migration inhibitory factor, activin, Fas ligand, protein S, and immunosuppressive cytokines such as interleukin (IL)-10 and transforming growth factor (TGF)-β, which play critical roles in regulating immune responses in the testes [1]–[3], [6].

If the BTB is severely damaged, then the autoimmunogenic spermatozoa leak out beyond the BTB, causing a continuous supply of autoantigens and resultant testicular inflammation. Damage of the BTB in one testis after an infection, biopsy, or surgery in the scrotal area has been reported to induce orchitis in the contralateral testis [7]. Experimental autoimmune orchitis (EAO), which can be induced by immunization of syngeneic testicular antigens, is a useful model for studying chronic testicular inflammation and infertility [8]. EAO has been induced in several animals, including monkeys, rats, and mice, and it is characterized by intratesticular infiltration of CD4^+^ T cells, CD8^+^ T cells, B cells, and plasma cells, followed by spermatogenic disturbance, apoptosis, and sloughing [8]. Recent studies revealed that CD4^+^ and CD8^+^ T cells producing interferon (IFN)-γ and IL-17, which are orchestrated by IL-12- and IL-23-producing macrophages and/or dendritic cells, are critically involved in the pathogenesis of EAO [9], [10].

The IL-6/IL-12 family cytokines IL-12, IL-23, IL-27, and IL-35 play critical roles in the regulation of various immune responses [11]–[15]. This family of cytokines has unique characteristics: they are heterodimers comprising two different subunits, and one subunit of the cytokine or cytokine receptor is sometimes shared with more than two cytokines. For instance, IL-12, IL-23, IL-27, and IL-35 are composed of p40 and p35, p40 and p19, Epstein-Barr virus−induced gene 3 (EBI3) and p28, and EBI3 and p35, respectively [11]–[14]. IL-12 and IL-23 play critical roles in the induction of helper T (Th)1 and Th17 differentiation, respectively [11], [12]. IL-27, with a receptor composed of gp130 and WSX-1, has both pro- and anti-inflammatory properties, including promotion of early Th1 differentiation, generation of IL-10-producing regulatory T cells (type 1 Treg cells), and suppression of Th2 and Th17 differentiation [12], [13]. IL-35, a novel anti-inflammatory cytokine that is produced by Foxp3^+^ Treg cells, suppresses cell proliferation and downregulates Th17 cell development [16], [17]. The IL-35 receptor consists of gp130, a common receptor for the IL-6 cytokine family, and IL-12 receptor (R) β2, which is one subunit of the IL-12 receptor [18]. Despite these numerous studies, the roles of IL-6/IL-12 family cytokines in the testicular immune privilege are not well understood.

In this study, we investigated whether IL-6/IL-12 family cytokines and their receptors are expressed in the testes and whether they play any role in the testicular immune privilege by using mice deficient in these subunits. Our results suggest that p35 helps in maintaining the testicular immune privilege, in part in an IL-35-dependent manner. This is the first report to our knowledge regarding the possible expression of IL-35 in the testicular macrophages and its implication in the immune privilege.

## Materials and Methods

### Ethics Statement

The animal study was approved by the institutional review board of Tokyo Medical University (S-22004, S-23043, S-24012, and S-25004).

### Mice

Wild-type (WT) C57BL/6 mice were purchased from SLC (Shizuoka, Japan). Mice deficient in EBI3 [19], p35 [20], IL-12Rβ2 [21], and IL-10 [22] in the C57BL/6 background were purchased from the Jackson Laboratory (Bar Harbor, ME, USA) and bred and maintained in the Laboratory Animal Center of Tokyo Medical University.

### Real-time RT-PCR Reaction

Total RNA was isolated from tissues using the TRIzol RNA extraction kit (Invitrogen, Carlsbad, CA, USA) and reverse-transcribed into cDNA by using the High-Capacity cDNA Archive Kit (Applied Biosystems, Foster City, CA, USA) according to the manufacturer’s instructions. Quantification of cDNA was performed by using SYBR Premix Ex Taq II (TaKaRa, Ohtsu, Japan) and Thermal Cycler Dice Real-Time System TP800 (TaKaRa). Glyceraldehyde-3-phosphate (GAPDH) was used as a housekeeping gene to normalize mRNA expression. Relative expression of real-time PCR products was determined by using the ΔΔCt method to compare target gene and GAPDH mRNA expression. Primers used in this study are listed in Table S1.

### ELISA

Testes were gently homogenized in PBS on ice and centrifuged. Resultant soluble supernatants were used to determine IFN-γ production levels by ELISA kit according to the manufacturer’s instructions (eBioscience, La Jolla, CA, USA).

### Flow Cytometry

Testicular interstitial cells were prepared as previously described [23], [24]. Briefly, testes were treated with type I collagenase (1 mg/ml, Wako Pure Chemical Industries, Ltd., Osaka, Japan) at 37°C for 30 min and resultant single suspension cells were separated with a 40%/70% discontinuous Percoll density gradient (GE Healthcare, Uppsala, Sweden). These cells were first stimulated with phorbol 12-myristate 13-acetate and ionocymin for 3 h in the presence of brefeldin A, stained with antibodies against cell surface antigens, fixed, and permeabilized using the staining buffer set according to the manufacturer’s instructions (eBioscience). Antibodies against cell surface antigens of CD4, CD8, CD45, B220 and F4/80 (Biolegend, San Diego, CA, USA, or eBioscience) were used after blocking nonspecific Fc binding with anti-CD16/CD32 (2.4G2, American Type Culture Collection, Manassas, VA, USA). These cells were then intracellularly stained with antibodies against IFN-γ, IL-17, and Foxp3 (Biolegend or eBioscience) and analyzed with the FACSCanto II flow cytometer (BD Biosciences). Data analysis was performed with the FlowJo 9.6.2 software (Tree Star, Ashland, OR), and the absolute number of positive cells per testis was calculated from percentages obtained by flow cytometric analysis and total number of interstitial cells.

### Immunohistochemical Detection of T and B cells

Testes or brains of mice were removed, placed in OCT compound (Miles Laboratories, Naperville, IL, USA), frozen in liquid nitrogen, and stored at −80°C until they were used. Sections (5 µm thick) were cut with a cryostat (CM1900; Leica, Wetzlar, Germany) and then fixed in ethanol. After blocking with Block Ace (Yukijirushi, Hokkaido, Japan), the serial sections were incubated with rat anti-mouse CD4 (clone: H129.19, ×200; BD Biosciences), CD8 (clone: 53–6.72, ×50; BD Biosciences), or B220 (clone: RA3-6B2, ×200; BD Biosciences) monoclonal antibodies, followed by incubation with rabbit anti-rat IgG (Vector Labs, Burlingame, CA, USA). Immunoreactive cells were visualized with a Vectastain ABC Kit (Vector Labs) with 3,3′-diaminobenzidine as the chromogen. Sections processed with phosphate-buffered saline instead of the primary antibodies were used as negative controls. These sections were counterstained with methyl green or hematoxylin. The histopathological changes were evaluated by counting the number of infiltrating lymphocytes per square millimeter of testicular tissue. The sections were analyzed by using light microscopy at ×200 magnification. Twenty 1-mm^2^ areas were randomly examined, and more than 200 round or oval seminiferous tubules were counted in each testis. The interstitial areas were determined using Image J version 1.45l (National Institutes of Health, Bethesda, MD, USA).

### Immunohistochemical Detection of Serum Autoantibodies

For detection of serum autoantibodies, testes of WT mice were placed in OCT compound, frozen in liquid nitrogen, and stored at −80°C until they were used. Sections (5 µm thick) were cut with a cryostat (CM1900), fixed in ethanol, and incubated with serially diluted serum samples from WT mice and mice deficient in EBI3, p35, and IL-12Rβ2, followed by incubation with horseradish peroxidase–conjugated goat anti-mouse IgG. Horseradish peroxidase–binding sites were detected with 3,3′-diaminobenzidine. These sections were counterstained with methyl green. The results were compared with those of sections background-stained and incubated with horseradish peroxidase–conjugated antibodies in the absence of primary antibodies.

### Histochemical Detection

Testes, eyes or brains were removed, fixed with Bouin solution, and embedded in plastic (Technovit 7100; Kulzer & Co., Wehrheim, Germany) without cutting the organs to avoid artificial damage to the tissue. Sections (5 µm thick) were obtained at 15- to 20-µm intervals and stained with hematoxylin and eosin for light microscopic observation. More than 100 round or oval seminiferous tubules were counted in each mouse, and the percentage of the seminiferous tubules showing the disappearance of mature germ cells and desquamation of germinal epithelium was calculated.

### Immunofluorescence Detection

Testes of WT mice were placed in OCT compound, frozen in liquid nitrogen, and stored at −80°C until they were used. Sections (5 µm thick) were cut with a cryostat (CM1900) and fixed in acetone. After blocking with Block Ace (Yukijirushi), the serial sections were incubated with rat anti-mouse EBI3 (clone: DNT27, ×100; Abcam, Cambridge, MA, USA), F4/80 (clone: A3–1, ×500; Abcam), rabbit anti-mouse CD163 (×200; Santa Cruz Biotechnology, Santa Cruz, CA, USA), p35 (clone: EP5737, ×500; Epitomics, Burlingame, CA, USA), anti-IL-12Rβ2 (×200; Bioss, Woburn, MA, USA), GATA1 (clone: N6, ×200; Santa Cruz Biotechnology), and Foxp3 (clone: H-190, ×200; Santa Cruz Biotechnology). After washing, the sections were incubated with Alexa 488–conjugated goat anti-rabbit IgG (Invitrogen) or Alexa 546–conjugated goat anti-rat IgG (Invitrogen). Finally, the sections were counterstained with DAPI (DAKO Inc., Glostrup, Denmark). Sections processed with phosphate-buffered saline instead of the primary antibodies were used as negative controls. Fluorescence was examined using Zeiss confocal laser-scanning microscopes (LSM510 and LSM510 META, Carl Zeiss Microscopy, Jena, Germany) with ×20 objectives. The images were corrected for brightness and contrast using a Zeiss LSM Image Browser, Adobe Illustrator 9.0 (Adobe, San Jose, CA, USA), and Adobe Photoshop 7.0. When the primary antibodies were omitted from immunofluorescent staining, no immunoreactivity was detected.

### Statistical Analysis

Data are expressed as mean ± SD. ANOVA and the Tukey post-hoc test were used for statistical analysis. Differences were considered to be statistically significant at *P*<0.05.

## Results

### Increased mRNA Expression of EBI3 but Decreased mRNA Expression of p40 and WSX-1 in Testes of Adult WT Mice

**Figure 1:**
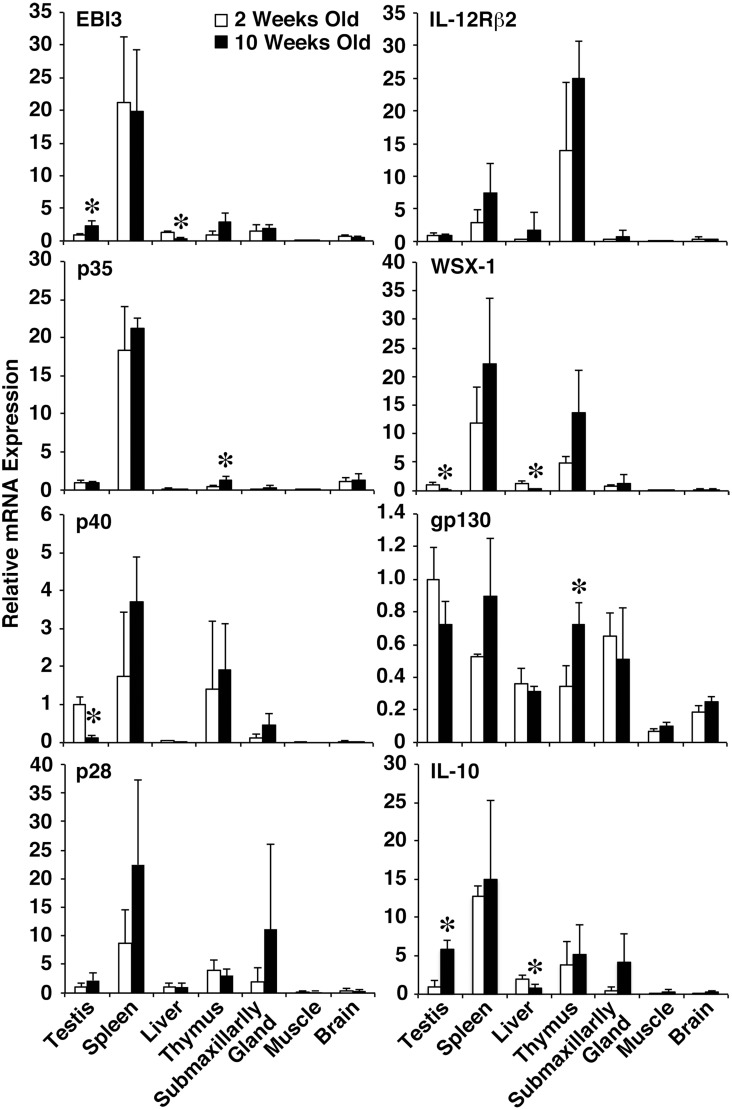
Increased mRNA expression of EBI3 and decreased mRNA expression of p40 and WSX-1 in testes of adult WT mice. Total RNA was prepared from various tissues of 2- and 10-week-old WT mice (*n* = 3 per group) and subjected to real-time RT-PCR using various sets of primers. Ratio of relative mRNA expression to the mRNA expression level in testes of 2-week-old mice was calculated. Data are shown as mean ± SD. *P<0.05 compared with 2-week-old mice. Similar results were obtained in two independent experiments.

To investigate the role of the IL-6/IL-12 family cytokines in maintaining the testicular immune privilege, we first examined the mRNA expression level of these cytokine subunits and their receptor subunits using WT mice. Total RNA was prepared from various organs of 2- and 10-week-old WT mice and subjected to real-time RT-PCR reaction using various sets of primers for these subunits. EBI3 mRNA expression was detected in testes at levels similar to those in the liver, thymus, and submaxillary gland (Fig. 1). Of note, EBI3 mRNA expression was significantly augmented in 10-week-old mice (2.3-fold increase) as compared with that in 2-week-old mice (Fig. 1). The p35 mRNA expression was also detected in the testes at levels similar to those in the thymus and brain, but the expression level was barely altered between 2- and 10-week-old mice (Fig. 1). Although the mRNA expression of p28, IL-12Rβ2, and gp130 was detected in the testes and barely altered between 2- and 10-week-old mice, the mRNA expression of p40 and WSX-1 was significantly reduced in 10-week-old mice (0.13-fold decrease and 0.25-fold decrease, respectively) compared with 2-week-old mice (Fig. 1). Consistent with constitutive production of IL-10 in the testicular CD163^+^ resident macrophages, as reported previously [25], greatly enhanced mRNA expression of IL-10 was detected in the testes of 10-week-old WT mice (5.9-fold increase) as compared with 2-week-old mice (Fig. 1). These results suggest that the mRNA expression of EBI3 and IL-10 is increased but the mRNA expression of p40 and WSX-1 is markedly decreased in adult testes, implying important roles for IL-35 and IL-10 in adult testes.

### Enhanced Infiltration of Lymphocytes in Testes of Mice Deficient in EBI3, p35, and IL-12Rβ2

**Figure 2:**
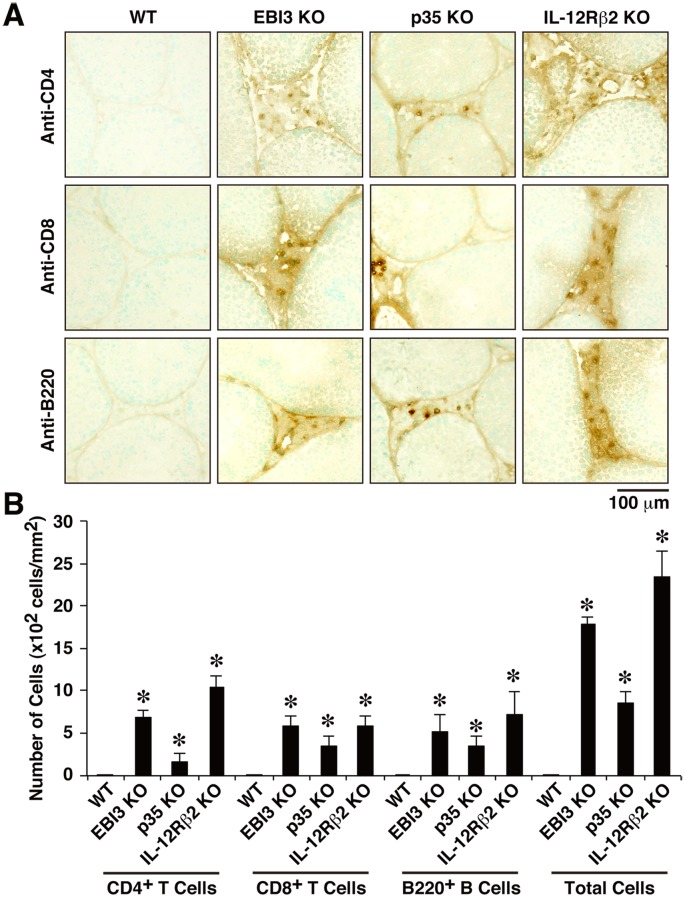
Enhanced infiltration of lymphocytes in testes of mice deficient in EBI3, p35, and IL-12Rβ2. (A) Cryostat sections of testes of WT mice and deficient mice (12 weeks old; *n* = 5 per group) were immunohistochemically stained with anti-CD4, anti-CD8, or anti-B220. The sections were also counterstained with methyl green. Representative histology images are shown. Positive cells are shown as gray spots. Dashed line indicates basal lamina of the seminiferous tubules. (B) The histopathological changes were evaluated by counting the number of infiltrating lymphocytes per square millimeter of testicular interstitium. Data are shown as mean ± SD. *P<0.05. Similar results were obtained in more than two independent experiments. KO, knockout.

In the following studies, therefore, we mainly focused on IL-35 in the testes. We first used mice deficient in EBI3, p35, and IL-12Rβ2 to examine their effects on the histopathological changes in the testes. Mice deficient in gp130 are not available, because these mice are embryonic-lethal [26]. Sections of testes from WT mice and from mice deficient in EBI3, p35, and IL-12Rβ2 were stained with hematoxylin and eosin. Increased infiltration of lymphocytes was observed in the testes of deficient mice, but not in those of 12-week-old WT mice (data not shown). To identify the cell type and the number of infiltrating lymphocytes, cryostat sections of testes were immunohistochemically stained with anti-CD4, anti-CD8, and anti-B220. CD4^+^ T cells, CD8^+^ T cells, and B cells were detected in the interstitium around the seminiferous tubules in the testes of all mice deficient in EBI3, p35, and IL-12Rβ2, but not in WT mice (Fig. 2A). The number of infiltrating lymphocytes in the testes of deficient mice was much higher than that in WT mice (Fig. 2B). Similar tendency was observed by quantitation of respective cell numbers per testis with a flow cytometer, although some of them failed to reach statistically significant results probably due to the small number of mice used (Fig. S1). Thus, lack of EBI3, p35, and IL-12Rβ2 induced enhanced infiltration of lymphocytes, including CD4^+^ T cells, CD8^+^ T cells, and B cells, in the interstitium of the testes.

### Augmented IFN-γ Expression and Autoantibody Production in Mice Deficient in EBI3, p35, and IL-12Rβ2

**Figure 3:**
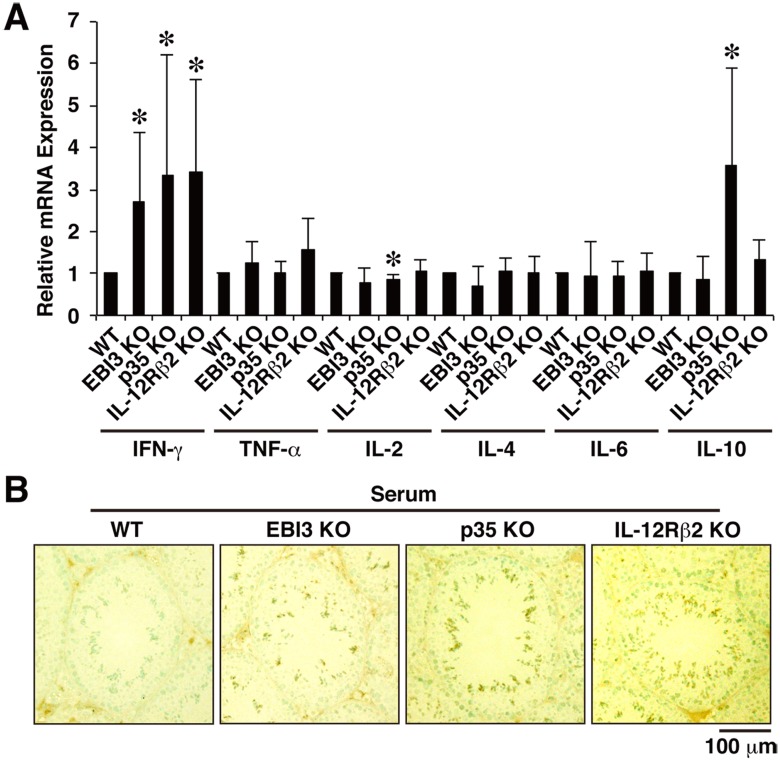
Augmented IFN-γ expression and autoantibody production in mice deficient in EBI3, p35, and IL-12Rβ2. (A) Total RNA was prepared from testes of WT mice and deficient mice (12 weeks old; *n* = 5 per group) and subjected to real-time RT-PCR using primers for various cytokines. Ratio of relative expression level of each cytokine in deficient mice to that in WT mice was calculated. Data are shown as mean ± SD. *P<0.05 compared with WT mice. (B) For detection of serum autoantibodies, cryostat sections of testes of WT mice (12 weeks old) were immunohistochemically stained with diluted serum samples (×50, *n* = 5 per group) obtained from WT mice and deficient mice (12 weeks old). The sections were also counterstained with methyl green. Representative histology images are shown. Positive cells are shown as dark brown spots. Similar results were obtained in three independent experiments.

To explore whether the inflammation, such as that caused by enhanced infiltration of lymphocytes, may cause any damage to the testis, we next examined mRNA expression levels of possible effector cytokines, including IFN-γ, tumor necrosis factor (TNF)-α, IL-2, IL-4, IL-6, and IL-10. Among these cytokines, IFN-γ mRNA expression significantly increased approximately three-fold in the testes of all mice deficient in EBI3, p35, and IL-12Rβ2 compared with WT mice (Fig. 3A). In addition, IL-10 mRNA expression was significantly enhanced in the testes of p35-deficient mice. Consistent with the results, analyses using a flow cytometer also showed similar tendency that the numbers of Th1 cells and IFN-γ^+^CD8^+^ T cells, but not Treg cells, in the testes of mice deficient in EBI3, p35, and IL-12Rβ2 were increased compared with those in WT mice (Fig. S2A–C). Although the numbers of Th17 cells and IL-17^+^CD8^+^ T cells also seem to be increased in these deficient mice, their absolute numbers were much less than Th1 cells and IFN-γ^+^CD8^+^ T cells (Fig. S2B and C). Such differences were not seen in the spleens (Fig. S2A–C). However, IFN-γ production at protein levels, which was determined by ELISA, in the total soluble fractions of testes and in the serum of WT mice and these deficient mice was below the detection limit (data not shown).

We next examined whether mice deficient in EBI3, p35, and IL-12Rβ2 produce any autoantibodies against antigens in testes. Cryostat sections of testes of 12-week-old WT mice were immunohistochemically stained with serially diluted serum samples from WT mice and from the deficient mice. Serum from all these deficient mice, but not from WT mice, mainly bound to acrosomal regions of spermatids (Fig. 3B and S3). Serum titers of these deficient mice were determined to be roughly same and approximately 200 (Fig. S3). These results suggest that lack of EBI3, p35, and IL-12Rβ2 results in augmented IFN-γ expression in testes but not in spleens and production of autoantibodies against self antigens, including spermatids.

### Spermatogenic Disturbance in Mice Deficient in EBI3, p35, and IL-12Rβ2

**Figure 4:**
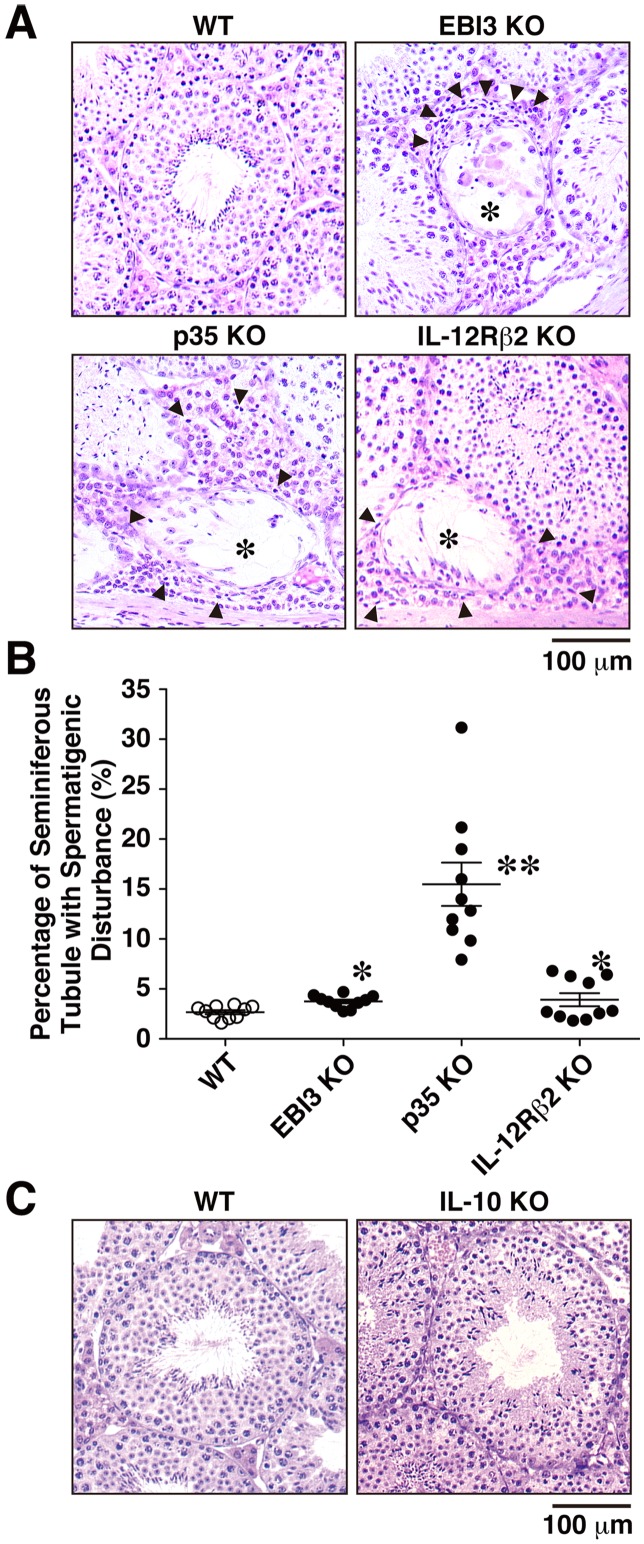
Spermatogenic disturbance in mice deficient in EBI3, p35, and IL-12Rβ2. (A) Sections of testes of WT mice and deficient mice (12 weeks old; *n* = 10 per group) were stained with hematoxylin and eosin. Representative histology images are shown. Infiltrating lymphocytes (arrow heads) were observed in the interstitium around the seminiferous tubules, especially with exhibition of spermatogenic disturbance (*), in deficient mice. (B) More than 100 round or oval seminiferous tubules were counted in each mouse, and the percentage of seminiferous tubules showing the disappearance of mature germ cells and desquamation of germinal epithelium was calculated. Data are shown as mean ± SD. *P<0.05 and **P<0.001 compared with WT mice, respectively. (C) Sections of testes of WT mice and IL-10-deficient mice were also similarly stained with hematoxylin and eosin. Representative histology images are shown. Similar results were obtained in two independent experiments.

We further investigated the influence of autoimmune-like inflammation, including enhanced infiltration of lymphocytes, increased IFN-γ expression, and autoantibody production. Sections of testes from WT mice and from the deficient mice were stained with hematoxylin and eosin. Increased infiltrating lymphocytes were observed in the interstitium around the seminiferous tubules in mice deficient in EBI3, p35, and IL-12Rβ2, but few were observed in WT mice. Intriguingly, most of these seminiferous tubules surrounded by infiltrating lymphocytes appeared to have impaired spermatogenesis, which is characterized by sloughing of germ cells (Fig. 4A). The percentage of seminiferous tubules exhibiting the spermatogenic disturbance was significantly higher in mice deficient in EBI3, p35, and IL-12Rβ2 than in WT mice (Fig. 4B). Among these deficient mice, p35-deficient mice had the most spermatogenic disturbance. Unexpectedly, however, no apparent histopathological abnormality was detected in the testes of IL-10–deficient mice (Fig. 4C). Therefore, lack of EBI3, p35, and IL-12Rβ2 but not IL-10 causes impaired spermatogenesis, possibly by infiltration of various lymphocytes.

### Expression Patterns of EBI3, p35, and IL-12Rβ2 in Testes

**Figure 5:**
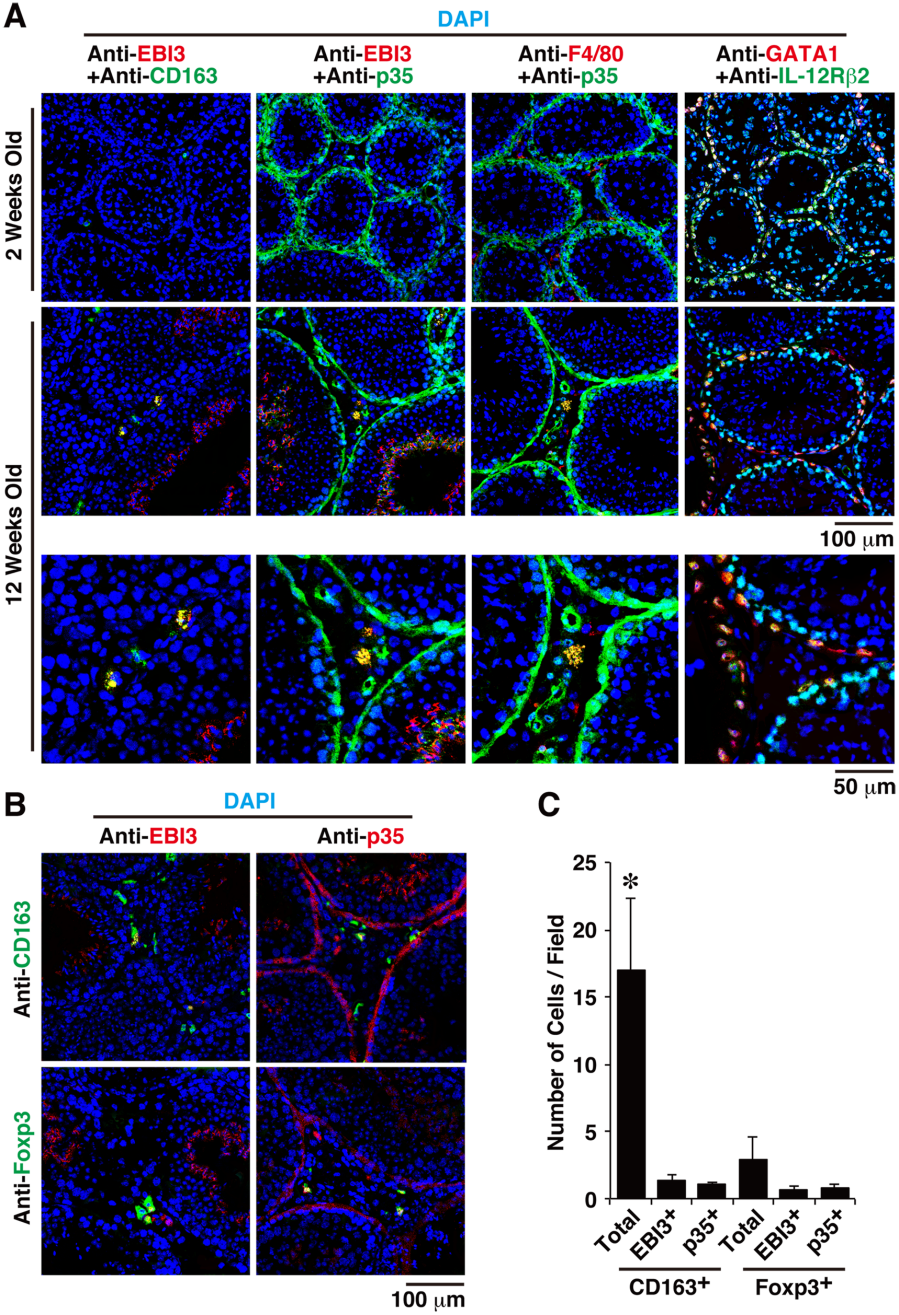
Expression patterns of EBI3, p35, and IL-12Rβ2 in testes. (A) Cryostat sections of testes of WT mice (2 and 12 weeks old; *n* = 5 per group) were immunohistochemically stained with anti-EBI3, anti-CD163, anti-p35, anti-F4/80, anti-GATA1, anti-IL-12Rβ2, and DAPI. Representative confocal merged images are shown. F4/80, CD163, and GATA1 are specific markers for whole macrophages, a subset of resident macrophages and Sertoli cells, respectively. Similar results were obtained in more than two independent experiments. (B) Cryostat sections of testes of WT mice (12 weeks old; *n* = 3 per group) were immunohistochemically stained with anti-EBI3, anti-p35, anti-CD163, anti-Foxp3, and DAPI. Representative confocal merged images are shown. (C) The number of positively stained cells was counted in multiple fields of microscope and calculated as cells per field. Data are shown as mean ± SD. *P<0.05 compared with Foxp3^+^ cells.

Because all mice deficient in EBI3, p35, and IL-12Rβ2 showed autoimmune-like inflammation, we tried to identify which types of cells express these molecules, perhaps including IL-35, in the testes. Cryostat sections of testes of 2- and 12-week-old WT mice were immunohistochemically stained with anti-EBI3, anti-p35, anti-CD163, anti-F4/80, anti-Foxp3, and DAPI, after specificities of these antibodies (Fig. S4) and negligible influences of autofluorescence in the sections (Fig. S5) were confirmed. Although EBI3-positive cells were rarely observed in the testes of 2-week-old mice, EBI3 expression was detected in the acrosomal regions of spermatids in seminiferous tubules of 12-week-old mice (Fig. 5A), which is consistent with the increased EBI3 mRNA expression in adult testes (Fig. 1). Of note, the cytoplasm of approximately 10% of macrophages positive for CD163 (Fig. 5C), which is a marker for resident macrophages [25], in testicular interstitium was also positive for EBI3 in 12-week-old mice (Fig. 5A). The p35 expression was similarly but slightly detected in the acrosomal regions of spermatids in seminiferous tubules, as was EBI3 expression, although different parts of the acrosomal regions appeared positive for p35 and EBI3 (Fig. 5A). Moreover, some EBI3^+^ and F4/80^+^ macrophages in the testicular interstitium were positive for p35 in 12-week-old mice but not in 2-week-old mice (Fig. 5A). In contrast, the cytoplasm of endothelial cells and peritubular cells were also positive for p35 in both 2- and 12-week-old mice (Fig. 5A). Some Foxp3^+^ Treg cells were also seen in the testicular interstitium (Fig. 5B) as reported before [27], and the cell numbers of Foxp3^+^EBI3^+^ or Foxp3^+^p35^+^ Treg cells were similar to or slightly less than those of CD163^+^EBI3^+^ or CD163^+^p35^+^ macrophages (Fig. 5C). In addition, immunohistochemical analysis of sections of testes from WT mice using anti-IL-12Rβ2 and anti-GATA1 revealed that IL-12Rβ2 expression was colocalized in GATA1^+^ cells (Fig. 5A). GATA1 is a maker for Sertoli cells [28], which favor the local immune privilege [29]. These results indicate that endothelial cells and peritubular cells in the interstitium were constitutively positive for p35, and that some CD163^+^ resident macrophages were positive for EBI3 and p35, possibly producing immunosuppressive IL-35, in addition to Treg cells in the testicular interstitial compartment of adult mice.

### No Impaired Histopathology in Eyes and Brains of Mice Deficient in EBI3 and p35

**Figure 6:**
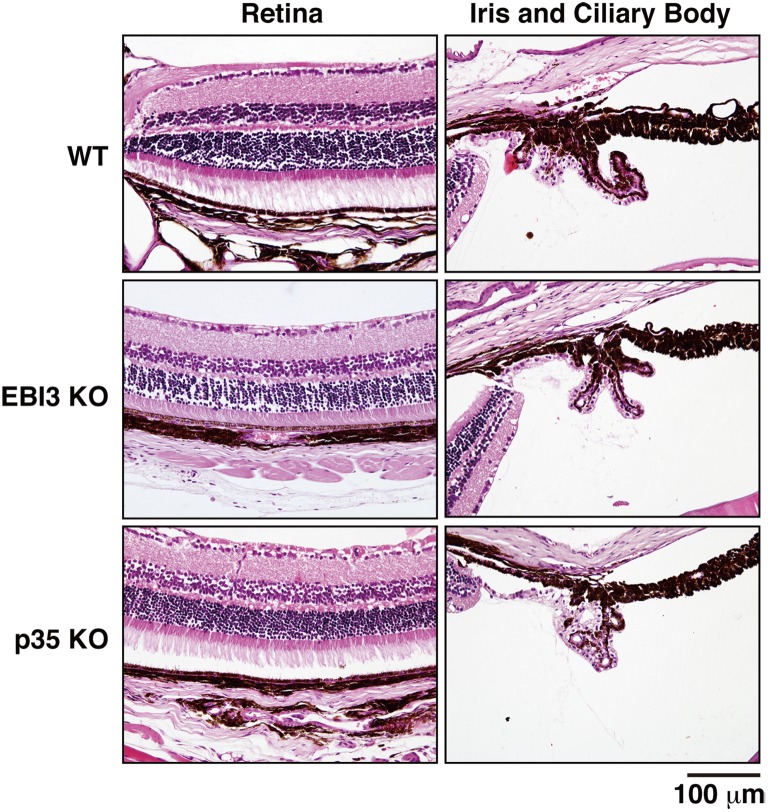
No impaired histopathology in eyes of mice deficient in EBI3 and p35. Sections of eyes of WT mice and deficient mice (12 weeks old; *n* = 3 per group) were stained with hematoxylin and eosin. Representative histology images are shown. No impaired histology in the retina, iris, and ciliary body was observed in the eyes of deficient mice compared with those of WT mice.

Finally, we investigated whether IL-35 also contributes in other sites of immune privilege, such as the eye and brain. Fluorescence angiographic analysis of eyes revealed that there was no abnormality in the eyes of 12-week-old mice deficient in EBI3 and p35 compared with those of WT mice (data not shown). Moreover, sections of eyes of these mice were stained with hematoxylin and eosin. No impaired histopathology in the retina, iris, or ciliary body was observed in the eyes of these deficient mice compared with those of WT mice (Fig. 6). Similarly, abnormal histopathology and infiltration of lymphocytes were not seen in the brains of these deficient mice (Fig. S6). These results suggest that lack of EBI3 and p35 does not affect the eyes and brains and, more specifically, contributes to induction of autoimmune-like inflammation in the testes, resulting in impaired spermatogenesis.

## Discussion

IL-35 has been demonstrated to inhibit autoimmune inflammation in various autoimmunity models, such as collagen-induced autoimmune arthritis [30], autoimmune demyelination in the central nervous system [31], and experimental colitis [17], [32]. Although secretion of bioactive IL-35 was reported in Treg cells [16], the contribution of IL-35 generated from tissues and cells other than Treg cells remains poorly understood. A recent study reported that human placental trophoblasts constitutively express and secrete IL-35, which might contribute to their suppressive capacity in maternal immune cells [33], and that conventional activated human T cells also secrete high levels of IL-35 [34]. Treg cells play critical roles in the maintenance of immune homeostasis and tolerance through multiple mechanisms, including (1) the secretion of immunosuppressive soluble factors, such as IL-10, TGF-β, and IL-35; (2) cell contact–mediated regulation via the high-affinity T-cell receptor and other costimulatory molecules, such as cytotoxic T-lymphocyte antigen 4 and glucocorticoid-induced TNF receptor–related protein; and (3) cytolytic activity [35], [36]. Genetic or physical ablation of the Treg population systemically causes spontaneous development of autoimmune diseases and enhanced inflammation, indicating that Treg cells play a central role in the control of systemic immune tolerance [35], [36]. Since no similar spontaneous development of aberrant inflammation has been reported in any organs of mice deficient in EBI3 and p35 [19], [20], except for the testes, as demonstrated here, the contribution of IL-35 to the suppressive function of Treg cells might be limited. It is currently considered that both systemic immune tolerance and local immunosuppression are important to maintain the immune privilege status [3]. Indeed, there were Foxp3^+^ cells in the testicular interstitium as reported previously [27], and some of them are positive for EBI3 and p35, (Fig. 5B and C). However, macrophages, which abundantly reside in the interstitium and represent a major population among immune cells in testes [37], were previously reported to have tissue-specific characteristics with a reduced capacity to produce inflammatory molecules compared with macrophages from other tissues and also with immunosuppressive functions [38], [39] as described below. Intriguingly, we found that the CD163^+^ macrophages in testes are also positive for p35 and EBI3 possibly producing IL-35 under physiological conditions as Treg cells (Fig. 5). Neither obvious inflammation was seen in the eyes and brains of EBI3 KO, p35 KO, and IL-12Rβ2 KO mice (Fig. 6 and Fig. S6), nor increased number of IFN-γ^+^CD4^+^/CD8^+^ T cells was observed in the spleens of these KO mice (Fig. S2A–C). Since the inflammation is thus locally seen only in testes and not systemic, the testicular macrophages are highly likely to specifically play a critical role in maintaining the immune privilege. Therefore, although Treg cells may also play a role in it to control the systemic immune tolerance as previously suggested [40], [41], the testicular macrophages would specifically contribute to maintaining the immune privilege status in testes by controlling the local immunosuppression possibly through IL-35. Moreover, because endothelial cells and peritubular cells in the interstitium were also highly positive for p35 but not for EBI3 (Fig. 5A) and p35-deficient mice showed the worst exacerbation of spermatogenic disturbance (Fig. 4B), an IL-35-independent but p35-dependent mechanism may also contribute to maintaining the immune privilege. Further studies are necessary to clarify the ability of testicular macrophages to produce functional IL-35 and the molecular mechanism by which p35 also controls the immune privilege in an IL-35-independent manner.

A developmental study revealed that the accumulation of macrophages in the testis is an acquired trait that starts and ends before puberty [42]. Under physiological conditions, macrophages represent a major population among immune cells in the interstitial space of the testis and occupy approximately 20% to 25% of the interstitial cells of rats [37]. As a major immune cell population, the testicular macrophages are believed to be critical in maintaining the testicular immune environment, particularly its immune privilege properties [3], [38]. Rat testicular macrophages are classified into two main populations, which are designated “resident” and “newly arrived” according to the differential expression of surface scavenger receptor CD163 [25]. The CD163^+^ resident macrophages, which represent approximately 80% of all testicular macrophages, constitutively produce immunosuppressive IL-10 and are poor stimulators of T-cell proliferation, indicating that they contribute to the maintenance of the testicular immune privilege [3], [38]. However, CD163^−^ monocyte–like newly arrived macrophages, which have a pro-inflammatory phenotype and express IL-1β, TNF-α, IL-6, activin A, inducible nitric oxide synthase, and other inflammatory factors, infiltrate into the testes in response to lipopolysaccharide, resulting in systemic inflammation. Consistent with constitutive production of IL-10 in the testicular CD163^+^ macrophages, greatly enhanced mRNA expression of IL-10 was detected in the testes of 10-week-old adult WT mice as compared with that of 2-week-old mice (Fig. 1). Surprisingly, however, no apparent histopathological abnormality was detected in the testes of IL-10-deficient mice (Fig. 4C). Although increased IL-10 mRNA expression was observed in the testes of p35-deficient mice (Fig. 3A), this might have resulted from the compensatory mechanism in response to the severe spermatogenic disturbance in these mice (Fig. 4A and B). Collectively, these findings indicate that the testicular CD163^+^ resident macrophages may contribute to maintaining the immune privilege, possibly by producing IL-35 in addition to IL-10. Whether purified testicular CD163^+^ resident macrophages secrete functional IL-35 in vitro remains to be elucidated, although obtaining enough purified CD163^+^ macrophages from mouse testes might be problematic. In addition to CD163^+^ resident macrophages, different parts of the acrosomal regions of spermatids in seminiferous tubules were positive for EBI3 and p35 (Fig. 5A). Although cytokines such as TNF-α and TGF-β released from germ cells, particularly primary spermatocytes, were implicated in the junction restructuring of the BTB and apical ectoplasmic specialization [43], the physiological significance of the expression of EBI3 and p35 in spermatids remains unclear.

IL-12Rβ2-deficient mice older than age 1 year were previously reported to experience spontaneous systemic development of an autoimmune and lymphoproliferative disorder associated with enhanced susceptibility to tumor formation [44]. This disorder is characterized by immune complex–mediated glomerulonephritis, systemic IL-6 upregulation, and multi-organ lymphoid infiltrates with oligoclonal B-cell expansion, and its features appear different from those observed in the testes of IL-12Rβ2-deficient mice. Interestingly, immunohistochemical analysis of sections of testes from WT mice using anti-IL-12Rβ2 and anti-GATA1 revealed that IL-12Rβ2 expression was colocalized in Sertoli cells (Fig. 5A), which favor the local immune privilege [29]. Because the other IL-35 receptor subunit gp130 is ubiquitously expressed [26], these results imply that IL-35 may also act directly on Sertoli cells and may control the immune privilege. Further studies are necessary to elucidate the exact molecular mechanism whereby IL-35 or p35 regulate immune homeostasis and tolerance in the testis.

Testicular inflammation is inducible by immunization with testis antigens, as in EAO. The development of EAO is highly dependent on CD4^+^ T cells, and adoptive transfer of these pathogenic CD4^+^ T cells causes EAO in naive mice [8]. The histopathology of EAO is characterized by intratesticular infiltration of CD4^+^ T cells, CD8^+^ T cells, B cells, and plasma cells with enhanced mRNA expression of IFN-γ and TNF-α and production of autoantibodies against only the acrosomal region of spermatids, followed by spermatogenic disturbance [45]–[47]. These features are similar to those of the autoimmune-like inflammation that spontaneously develops in mice deficient in EBI3, p35, and IL-12Rβ2, as shown here. A previous study demonstrated that local injection of IFN-γ into the testis in vivo induced a direct cytotoxic effect on spermatogenic cells, indicating that IFN-γ is harmful to the seminiferous epithelium and results in spermatogenic disturbance [48]. In addition, IFN-γ increased the expression of Fas in membrane-bound and soluble forms of Sertoli cells and made them susceptible to Fas ligand–mediated cytotoxicity in the seminiferous epithelium [49]. We observed augmented IFN-γ expression in the testes of mice deficient in EBI3, p35, and IL-12Rβ2 (Fig. 3A). These deficient mice cannot produce immunosuppressive IL-35 from testicular resident macrophages or respond to it, and p35-deficient mice cannot exert IL-35-independent but p35-dependent immunosuppressive mechanism. Therefore, these mice do not maintain immune homeostasis and tolerance, resulting in enhanced infiltration of lymphocytes into the interstitium of the testis with enhanced IFN-γ expression and autoantibody production. The locally produced IFN-γ then damages the seminiferous tubules, possibly through Fas–Fas ligand interaction, which eventually causes aspermatogenesis. Although this scenario is highly conceivable, further studies are necessary to prove it. Regarding fertility, however, all of deficient mice in EBI3, p35, and IL-12Rβ2 still seemed to be fully fertile, even at age 16 weeks (data not shown). Moreover, testis weight as a percentage of body weight of 8-week-old mice deficient in EBI3, p35, and IL-12Rβ2 was similar to that of WT mice, and no difference was detected between the groups even at 16 weeks of age (Fig. S7). It was previously demonstrated that in an EAO model immunization with the higher amounts of testicular antigens induces the more severe inflammation, which well correlates with the infertility [50]. Therefore, the lack of IL-35 or p35 does not seem to be enough to induce inflammation resulting in infertility or testis weight loss.

Evidence supports the idea that the maintenance of immune privilege status in the testes is controlled by multiple mechanisms, including the sequestration of antigens and antibodies from the immune system by the BTB, the immunosuppressive properties of local cells, and the production of paracrine and endocrine factors [1]–[3]. Our findings indicate that p35 may contribute to maintaining the testicular immune privilege, in part in an IL-35-dependent manner. Because autoimmunity and inflammation are important etiological factors for male infertility, further studies of immunoregulation in the testes will provide important insight regarding interventions for this condition.

## Supporting Information

Figure S1 **Enhanced infiltration of lymphocytes in testes of mice deficient in EBI3, p35, and IL-12Rβ2.** Testicular interstitial cells in testes of WT mice and deficient mice (12 weeks old; *n* = 3 per group) were stained with anti-CD4, anti-CD8, or anti-B220 together with anti-CD45 pan leukocytes, and the numbers of CD45^+^CD4^+^ cells, CD45^+^CD8^+^ cells, CD45^+^B220^+^ cells, and total CD45^+^ cells were counted by a flow cytometer. Data are shown as mean ± SD. *P<0.05 compared with WT mice. KO, knockout.
(TIF)

Figure S2 **Enhanced infiltration of Th1 cells in testes but not in spleens of mice deficient in EBI3, p35, and IL-12Rβ2.** (A) Testicular interstitial cells in testes and spleen cells of WT mice and deficient mice (12 weeks old; *n* = 3 per group) were intracellularly stained for detection of IFN-γ^+^CD4^+^ Th1, IL-17^+^CD4^+^ Th17 and Focp3^+^CD4^+^ Treg cells. Representative plots of the percentages of Th1, Th17 and Treg cells in testes and spleens are shown. (B) The numbers of these cells per testis or 10^5^ spleen cells were counted by a flow cytometer. Data are shown as mean ± SD. *P<0.05 compared with WT mice. (C) These cells were also intracellularly stained for detection of IFN-γ^+^CD8^+^ T cells and IL-17^+^CD8^+^ T cells, and their cell numbers were similarly counted. Data are shown as mean ± SD. *P<0.05 compared with WT mice.
(TIF)

Figure S3 **Autoantibody production in mice deficient in EBI3, p35, and IL-12Rβ2.** For detection of serum autoantibodies, cryostat sections of testes of WT mice (12 weeks old) were immunohistochemically stained with diluted serum samples (×50, ×200 and ×800, *n* = 5 per group) obtained from WT mice and deficient mice (12 weeks old). Representative histology images are shown. Positive cells are shown as dark gray spots.
(TIF)

Figure S4 **Specificities of antibodies against EBI3, p35, and IL-12Rβ2.** To confirm the specificities of antibodies used in this study, cryostat sections of testes of WT mice and respective deficient mice (12 weeks old) were immunohistochemically stained with anti-EBI3, anti-p35, and anti-IL-12Rβ2 together with DAPI. Representative confocal merged images are shown.
(TIF)

Figure S5 **Negligible influences of autofluorescence in the testis sections.** To confirm the influences of autofluorescence, cryostat sections of testes of WT mice (12 weeks old) were immunohistochemically stained with anti-EBI3, anti-CD163, anti-p35, anti-F4/80, and their control antibodies together with DAPI. Representative confocal merged images are shown.
(TIF)

Figure S6 **No impaired histopathology in brains of mice deficient in EBI3, p35, and IL-12Rβ2.** Sections of brains of WT mice and deficient mice (12 weeks old; *n* = 3 per group) were immunohistochemically stained with anti-CD4, anti-CD8, and anti-B220. The sections were also counterstained with hematoxylin. Representative histology images are shown. No impaired histology in the brains of deficient mice was observed compared with those of WT mice.
(TIF)

Figure S7 **No impaired testis weight in mice deficient in EBI3, p35, and IL-12Rβ2.** The whole body weight of each mouse (*n* = 3 per group), aged 8 or 16 weeks, was measured and the testes were removed and weighed. Testis weight as a percentage of body weight is shown. No significant difference was observed between WT mice and respective mice deficient in EBI3, p35, and IL-12Rβ2.
(TIF)

Table S1 **Primers used in this study.**
**(**DOCX**)**
