# Supplementary figures and images for: Contribution of IL-12/IL-35 Common Subunit p35 to Maintaining the Testicular Immune Privilege
Source: PLoS One. 2014 Apr 23;9(4):e96120. doi: 10.1371/journal.pone.0096120 (PMC3997559; doi:10.1371/journal.pone.0096120)

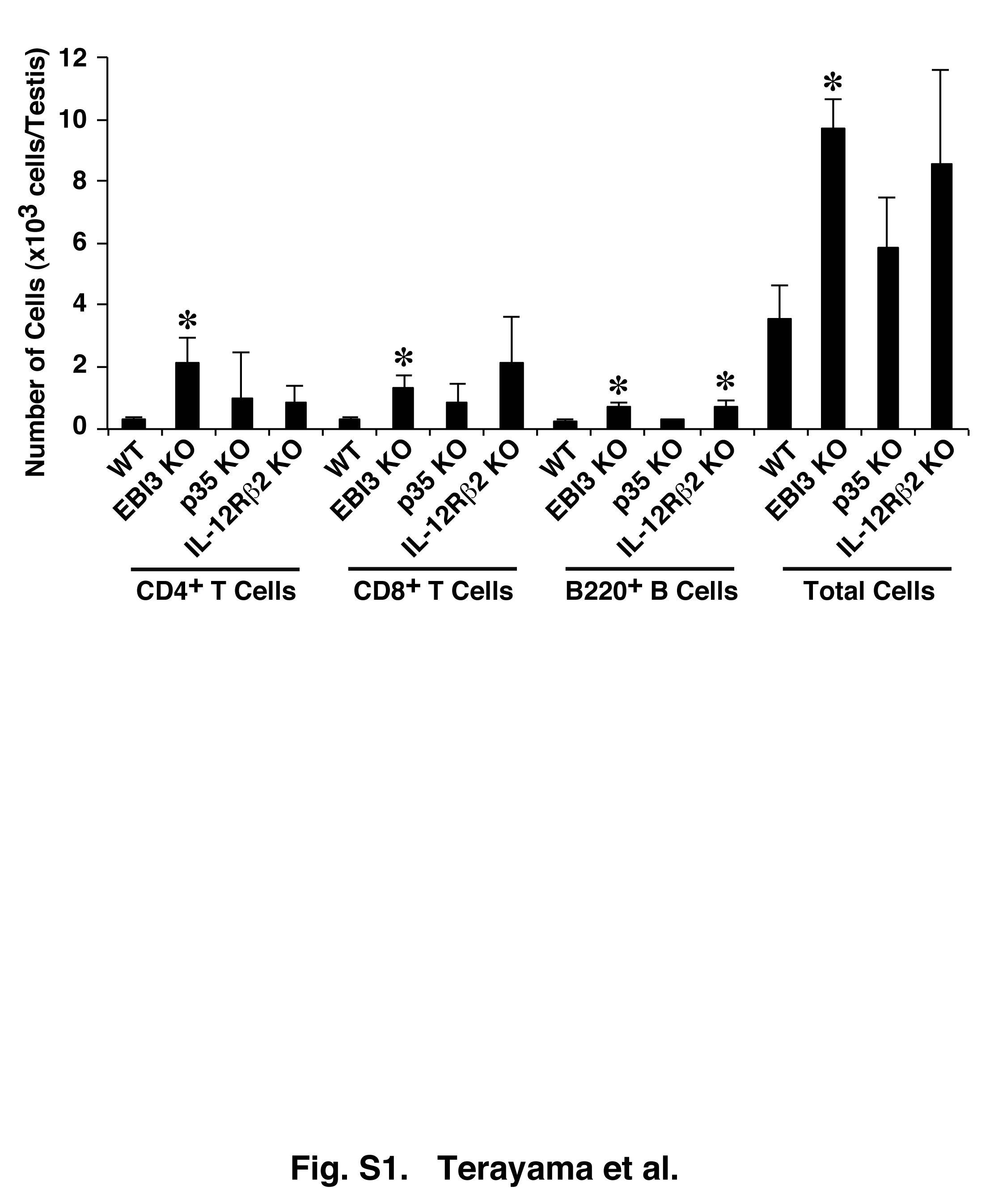

Supplement: Figure S1 — Enhanced infiltration of lymphocytes in testes of mice deficient in EBI3, p35, and IL-12Rβ2. Testicular interstitial cells in testes of WT mice and deficient mice (12 weeks old; n = 3 per group) were stained with anti-CD4, anti-CD8, or anti-B220 together with anti-CD45 pan leukocytes, and the numbers of CD45+CD4+ cells, CD45+CD8+ cells, CD45+B220+ cells, and total CD45+ cells were counted by a flow cytometer. Data are shown as mean ± SD. *P<0.05 compared with WT mice. KO, knockout. (TIF) [file pone.0096120.s001.tif]

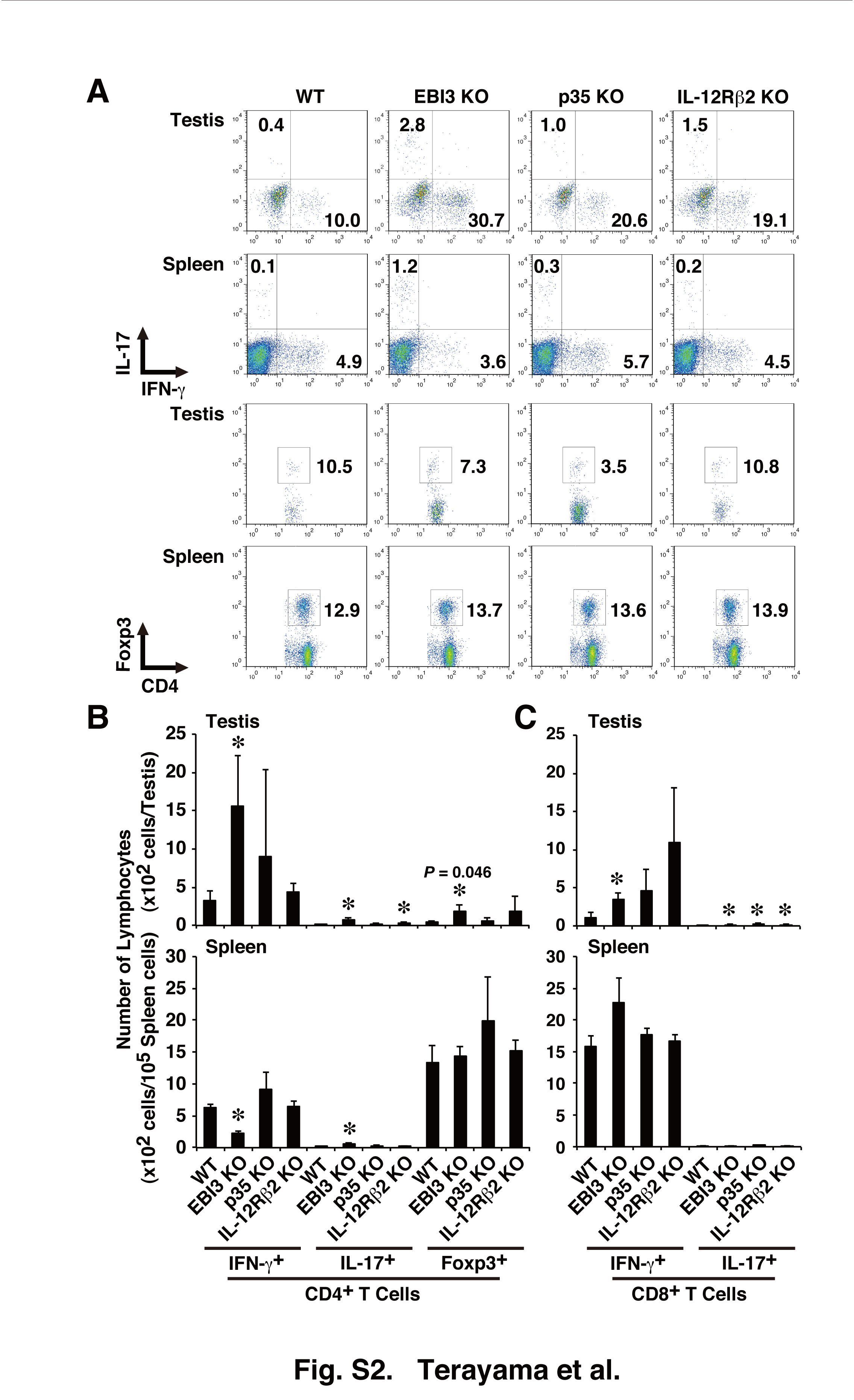

Supplement: Figure S2 — Enhanced infiltration of Th1 cells in testes but not in spleens of mice deficient in EBI3, p35, and IL-12Rβ2. (A) Testicular interstitial cells in testes and spleen cells of WT mice and deficient mice (12 weeks old; n = 3 per group) were intracellularly stained for detection of IFN-γ+CD4+ Th1, IL-17+CD4+ Th17 and Focp3+CD4+ Treg cells. Representative plots of the percentages of Th1, Th17 and Treg cells in testes and spleens are shown. (B) The numbers of these cells per testis or 105 spleen cells were counted by a flow cytometer. Data are shown as mean ± SD. *P<0.05 compared with WT mice. (C) These cells were also intracellularly stained for detection of IFN-γ+CD8+ T cells and IL-17+CD8+ T cells, and their cell numbers were similarly counted. Data are shown as mean ± SD. *P<0.05 compared with WT mice. (TIF) [file pone.0096120.s002.tif]

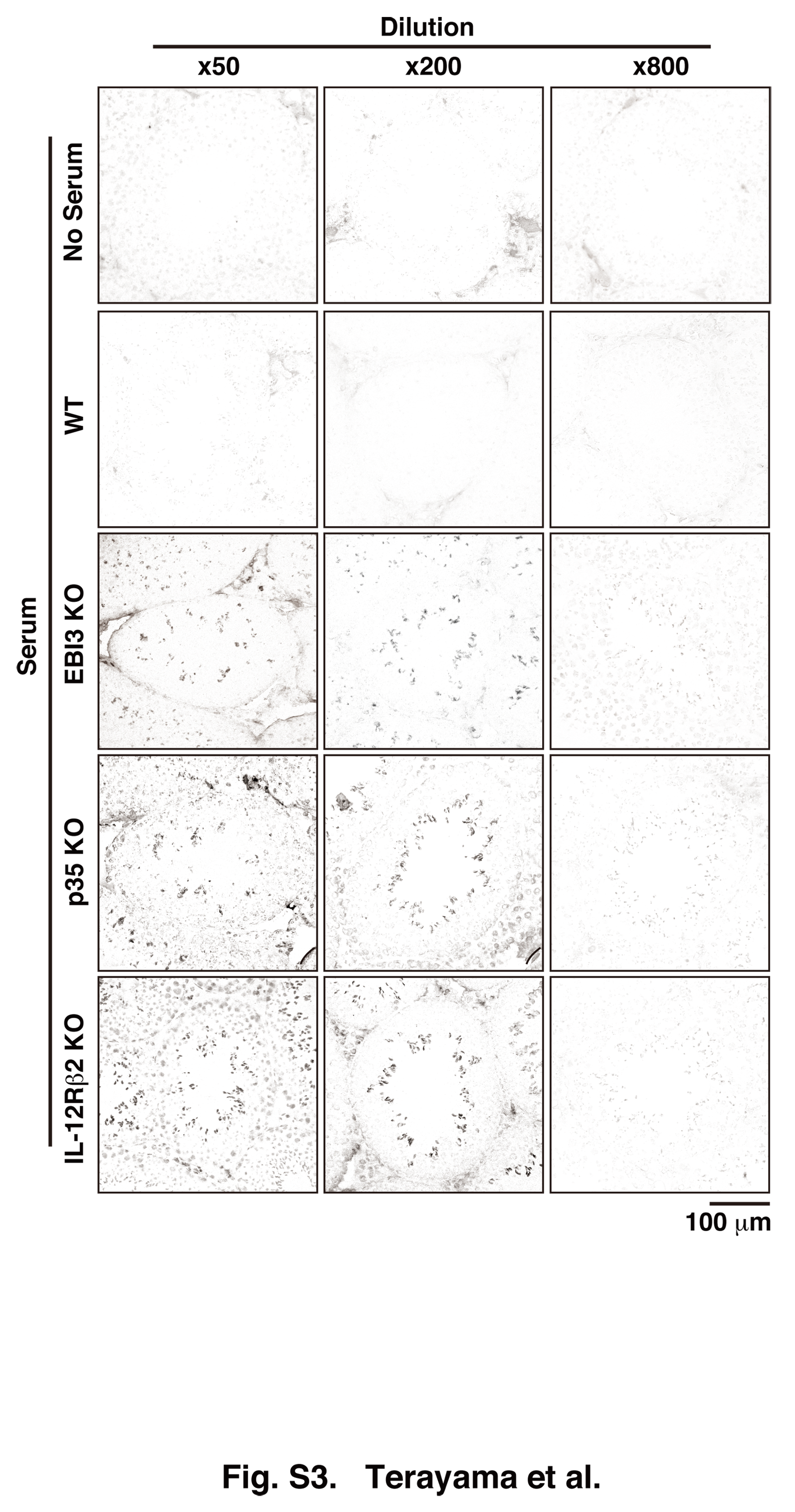

Supplement: Figure S3 — Autoantibody production in mice deficient in EBI3, p35, and IL-12Rβ2. For detection of serum autoantibodies, cryostat sections of testes of WT mice (12 weeks old) were immunohistochemically stained with diluted serum samples (×50, ×200 and ×800, n = 5 per group) obtained from WT mice and deficient mice (12 weeks old). Representative histology images are shown. Positive cells are shown as dark gray spots. (TIF) [file pone.0096120.s003.tif]

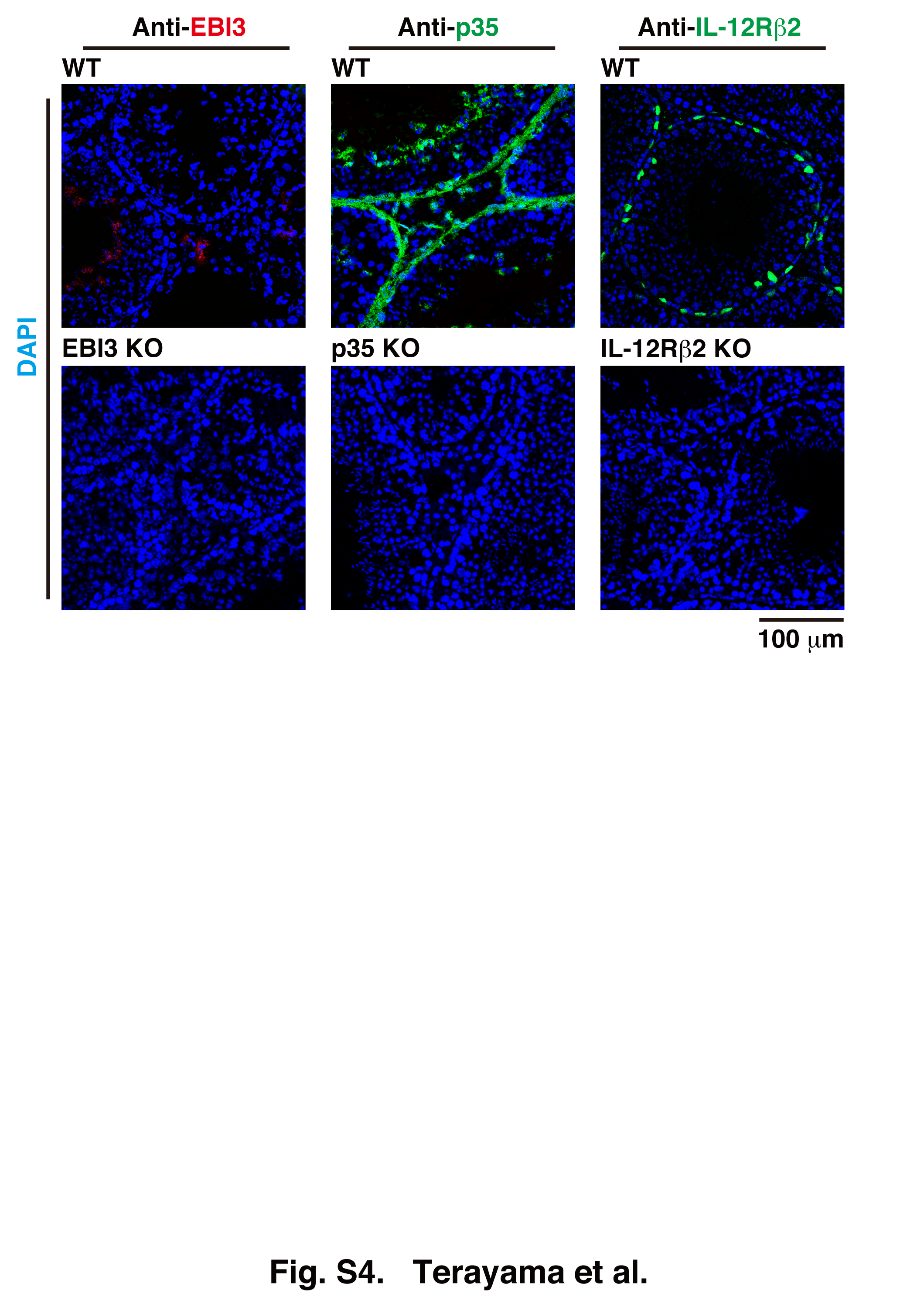

Supplement: Figure S4 — Specificities of antibodies against EBI3, p35, and IL-12Rβ2. To confirm the specificities of antibodies used in this study, cryostat sections of testes of WT mice and respective deficient mice (12 weeks old) were immunohistochemically stained with anti-EBI3, anti-p35, and anti-IL-12Rβ2 together with DAPI. Representative confocal merged images are shown. (TIF) [file pone.0096120.s004.tif]

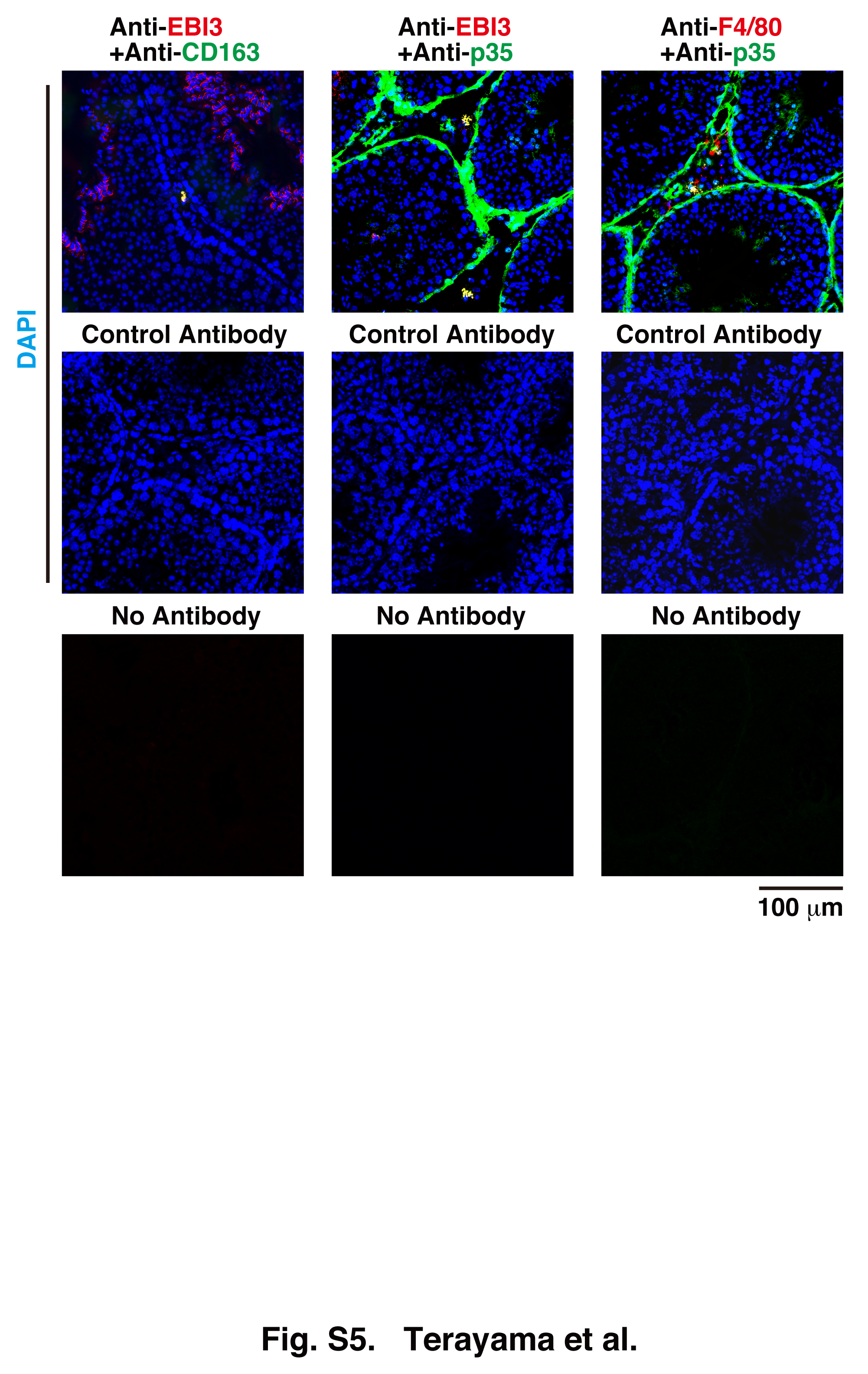

Supplement: Figure S5 — Negligible influences of autofluorescence in the testis sections. To confirm the influences of autofluorescence, cryostat sections of testes of WT mice (12 weeks old) were immunohistochemically stained with anti-EBI3, anti-CD163, anti-p35, anti-F4/80, and their control antibodies together with DAPI. Representative confocal merged images are shown. (TIF) [file pone.0096120.s005.tif]

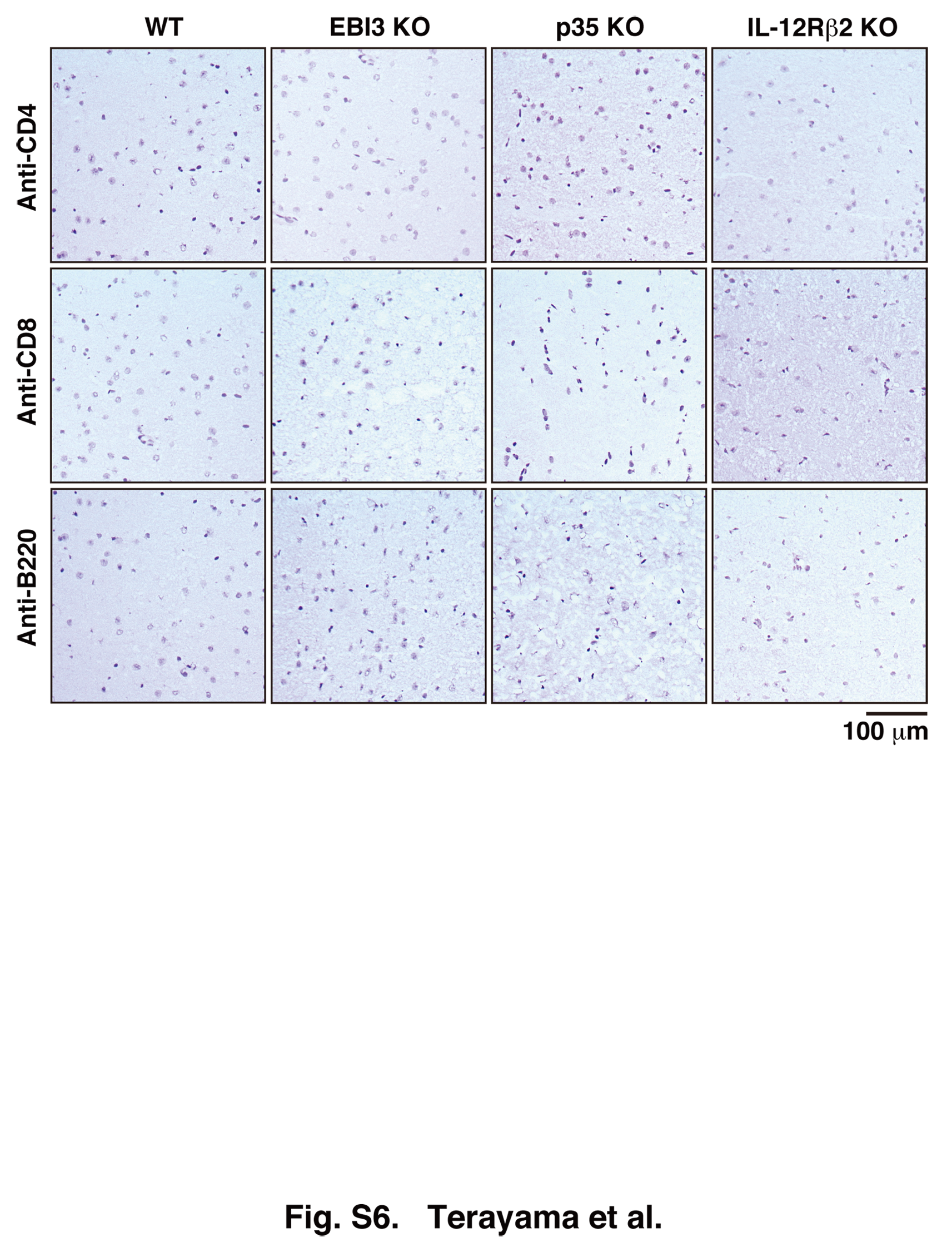

Supplement: Figure S6 — No impaired histopathology in brains of mice deficient in EBI3, p35, and IL-12Rβ2. Sections of brains of WT mice and deficient mice (12 weeks old; n = 3 per group) were immunohistochemically stained with anti-CD4, anti-CD8, and anti-B220. The sections were also counterstained with hematoxylin. Representative histology images are shown. No impaired histology in the brains of deficient mice was observed compared with those of WT mice. (TIF) [file pone.0096120.s006.tif]

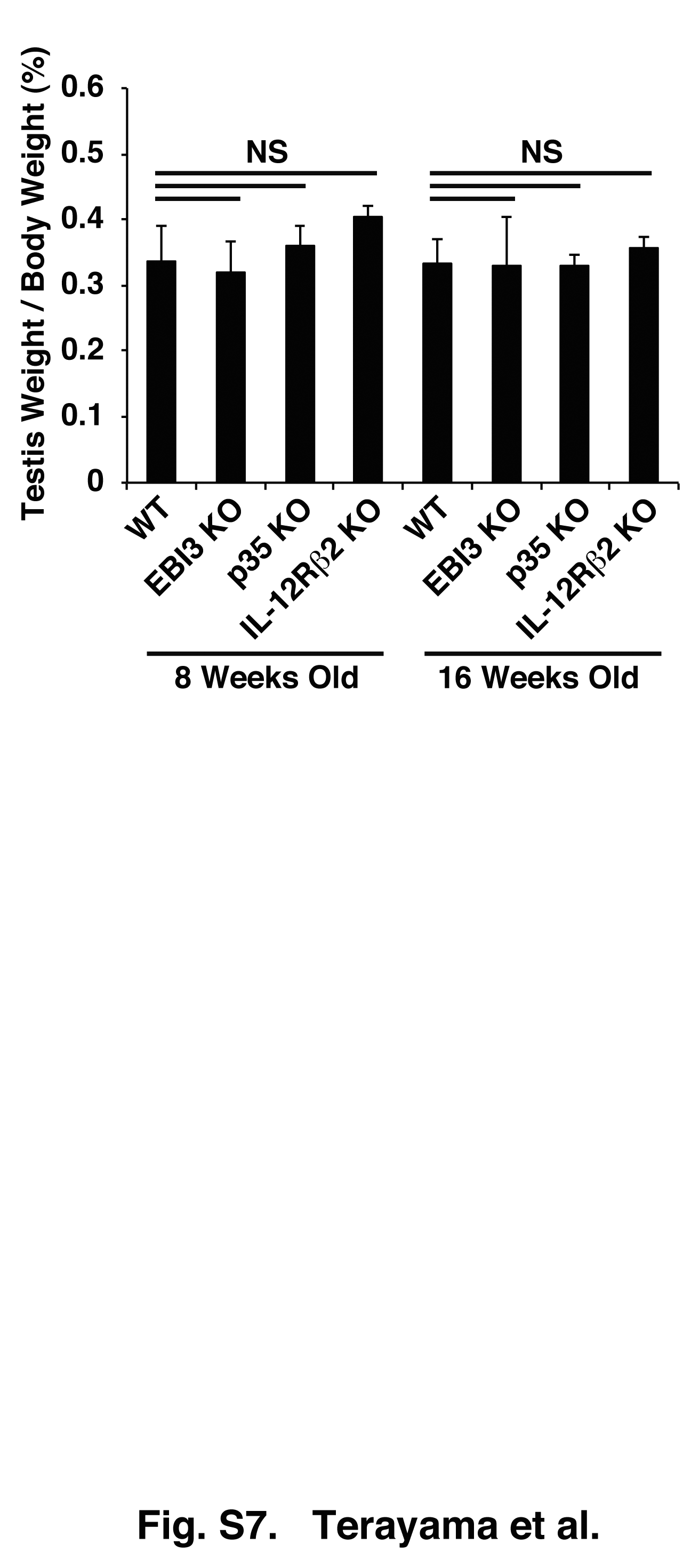

Supplement: Figure S7 — No impaired testis weight in mice deficient in EBI3, p35, and IL-12Rβ2. The whole body weight of each mouse (n = 3 per group), aged 8 or 16 weeks, was measured and the testes were removed and weighed. Testis weight as a percentage of body weight is shown. No significant difference was observed between WT mice and respective mice deficient in EBI3, p35, and IL-12Rβ2. (TIF) [file pone.0096120.s007.tif]
