# Supplementary material for: Contribution of IL-12/IL-35 Common Subunit p35 to Maintaining the Testicular Immune Privilege
Source: PLoS One. 2014 Apr 23;9(4):e96120. doi: 10.1371/journal.pone.0096120 (PMC3997559; doi:10.1371/journal.pone.0096120)
Supplement: Table S1 — Primers used in this study. (DOCX) [file pone.0096120.s008.docx]

**Supplementary Data**

**Table S1.** Primers used in this study.

| Name | Accession  Number | Direction | Sequence 5' to 3' |
| --- | --- | --- | --- |
| EBI3 | NM_015766.2 | forward  reverse | AGAGCCACAGAGCATGTCCAA  TGCACTCTGGGCTGGCTTAG |
| p35 | NM_001159424.1 | forward  reverse | CCGGTCCAGCATGTGTCAA  CAGGTTTCGGGACTGGCTAAGA |
| p40 | NM_008352.2 | forward  reverse | ACTCACATCTGCTGCTCCACAAG  CACGTGAACCGTCCGGAGTA |
| p28 | NM_145636.1 | forward  reverse | ACTCACATCTGCTGCTCCACAAG  CACGTGAACCGTCCGGAGTA |
| IL-12Rβ2 | NM_008354.3 | forward  reverse | GAGACTCGACAGCACAACCTGA  CTGTAGGCTGCTTATTGGATGTGA |
| WSX-1 | NM_016671.3 | forward  reverse | GGCCAGGCTACTCACTACACCTTC  GCAGTCTGGGTTTGACTGCTC |
| gp130 | NM_010560.3 | forward  reverse | GCACTTAGTTGGATGCAGTTTCAC  GCAAAGCATGTACAGTCTCACGA |
| IFN-γ | NM_008337.3 | forward  reverse | ATCTGGAGGAACTGGCAAAA  TTCAAGACTTCAAAGAGTCTGAGGTA |
| TNF-α | NM_013693.2 | forward  reverse | TCTTCTCATTCCTGCTTGTGG  TCTGGGCCATAGAACTGATGA |
| IL-2 | NM_008366.3 | forward  reverse | TTGACGGACCCCAAAAGAT  GAAGCTGGATGCTCTCATCTG |
| IL-4 | NR_027491.1 | forward  reverse | CATCGGCATTTTGAACGAG  CGAGCTCACTCTCTGTGGTG |
| IL-6 | NM_031168.1 | forward  reverse | GCTACCAAACTGGATATAATCAGGA  CCAGGTAGCTATGGTACTCCAGAA |
| IL-10 | NM_010548.2 | forward 1  reverse 1  forward 2  reverse 2 | GCCAGAGCCACATGCTCCTA  GATAAGGCTTGGCAACCCAAGTAA  CAGAGCCACATGCTCCTAGA  GTCCAGCTGGTCCTTTGTTT |
| GAPDH | NM_008084.2 | forward 1  reverse 1  forward 2  reverse 2 | GCACCGTCAAGGCTGAGAAC  TGGTGAAGACGCCAGTGGA  TTCAACGGCACAGTCAAGG  GTAGCCCAAGATGCCCTTC |
